# Uncertainty analysis of tumour absorbed dose calculations in molecular radiotherapy

**DOI:** 10.1186/s40658-020-00328-5

**Published:** 2020-10-12

**Authors:** Domenico Finocchiaro, Jonathan I. Gear, Federica Fioroni, Glenn D. Flux, Iain Murray, Gastone Castellani, Annibale Versari, Mauro Iori, Elisa Grassi

**Affiliations:** 1 Medical Physics Unit, Azienda Unità Sanitaria Locale di Reggio Emilia - IRCCS, Reggio Emilia, Italy; 2 Department of Physics and Astronomy, University of Bologna, Bologna, Italy; 3 The Royal Marsden NHS Foundation Trust & Institute of Cancer Research, Downs Road, Sutton, SM2 5PT UK; 4 Nuclear Medicine Unit, Azienda Unità Sanitaria Locale di Reggio Emilia - IRCCS, Reggio Emilia, Italy

**Keywords:** MRT, RPT, Radionuclide therapy, PRRT, Dosimetry, Accuracy, Uncertainty analysis

## Abstract

**Background:**

Internal dosimetry evaluation consists of a multi-step process ranging from imaging acquisition to absorbed dose calculations. Assessment of uncertainty is complicated and, for that reason, it is commonly ignored in clinical routine. However, it is essential for adequate interpretation of the results. Recently, the EANM published a practical guidance on uncertainty analysis for molecular radiotherapy based on the application of the law of propagation of uncertainty. In this study, we investigated the overall uncertainty on a sample of a patient following the EANM guidelines. The aim of this study was to provide an indication of the typical uncertainties that may be expected from performing dosimetry, to determine parameters that have the greatest effect on the accuracy of calculations and to consider the potential improvements that could be made if these effects were reduced.

**Results:**

Absorbed doses and the relative uncertainties were calculated for a sample of 49 patients and a total of 154 tumours. A wide range of relative absorbed dose uncertainty values was observed (14–102%). Uncertainties associated with each quantity along the absorbed dose calculation chain (i.e. volume, recovery coefficient, calibration factor, activity, time-activity curve fitting, time-integrated activity and absorbed dose) were estimated. An equation was derived to describe the relationship between the uncertainty in the absorbed dose and the volume. The largest source of error was the VOI delineation. By postulating different values of FWHM, the impact of the imaging system spatial resolution on the uncertainties was investigated.

**Discussion:**

To the best of our knowledge, this is the first analysis of uncertainty in molecular radiotherapy based on a cohort of clinical cases. Wide inter-lesion variability of absorbed dose uncertainty was observed. Hence, a proper assessment of the uncertainties associated with the calculations should be considered as a basic scientific standard. A model for a quick estimate of uncertainty without implementing the entire error propagation schema, which may be useful in clinical practice, was presented. Ameliorating spatial resolution may be in future the key factor for accurate absorbed dose assessment.

## Background

In recent decades, molecular radiotherapy (MRT) has been increasingly used for the treatment of neuroendocrine tumours (NETs). The use of somatostatin analogues labelled with radio-emitting isotopes has shown promising results [1–3], and it is expected that peptide receptor radionuclide therapy (PRRT) will become more widely used. Recently, the NETTER-1 trial [4] demonstrated that ^177^Lu-DOTATATE-PRRT significantly improved progression-free survival. It has also been demonstrated that absorbed doses delivered to healthy organs and tumours have large inter-patient variability [5–8]. Moreover, many studies have provided evidence of dose-effect correlations in PRRT [9–11]. For these reasons, groups from different hospitals and research institutes across Europe have proposed the use of dosimetry for PRRT in routine clinical practice [12]. Personalized medicine necessitates treatment to be optimized based on patient-specific dosimetry. Calculation of the absorbed doses delivered to organs at risk and tumours should ideally incorporate uncertainty analysis. This is particularly true in the case of tumour dosimetry that can be subjected to relatively high uncertainties due to the wider range of absorbed doses delivered and the lack of standardised S-factors [13, 14].

To date, investigations into uncertainties of absorbed dose calculations in MRT have been mainly based on phantom measurements or simulated data [15–18]. However, uncertainty evaluation should ideally be considered for each individual case. Furthermore, the majority of studies have focused on one or only a few aspects of MRT absorbed dose measurements (for example on the calibration of gamma cameras [19] or on activity quantification [20–22]). However, internal dosimetry evaluation consists of a multi-step process with a specific uncertainty associated with each step [23]. Consequently, each step should be included in the overall absorbed dose uncertainty calculation.

Recently, the EANM published practical guidance on uncertainty analysis for molecular radiotherapy absorbed dose calculations [24]. This guide provides a detailed schema to determine uncertainties based on the application of the law of propagation of uncertainty (LPU) and was designed to be implemented using standard resources available in every clinic offering MRT. The published EANM paper also reports a patient example to support readers for the implementation of the guidelines.

To the best of our knowledge, to date, there are no published data to address uncertainty analysis that includes every aspect of the dosimetry calculation chain on a sample of clinical cases.

In that context, this study shows the results of uncertainties in tumour absorbed dose calculations for a sample of patients treated at Azienda USL-IRCCS of Reggio Emilia (Italy). The scope of this paper is to give an indication of the typical uncertainties that may be expected from performing tumour dosimetry, to determine parameters that have the greatest effect on the accuracy of calculations and to consider the potential improvements that could be made if these effects were reduced.

## Materials and methods

### Patients

This study was carried out retrospectively on a sample of 49 patients enrolled in a clinical trial between 2016 and 2017 (EUDRACT 2015-005546-63), which received local institutional ethics committee approval at the Azienda USL-IRCCS of Reggio Emilia hospital.

All patients were affected by NETs and were treated with PRRT. According to the trial design, each patient underwent several ^177^Lu- and ^90^Y-DOTATOC administrations. The dosimetry was conducted at the first cycle of therapy after a therapeutic injection of ^177^Lu-DOTATOC. A mean value of 4.2 ± 0.9 GBq of ^177^Lu-DOTATOC was administered to patients. A maximum of 5 lesions was analysed for each patient, with a total of 154. The clinical trial was conducted before the Lutathera approval by EMA and FDA.

### Imaging

All examinations were performed using a hybrid Symbia T2 SPECT/CT (Siemens Healthineers, Germany). The SPECT gamma camera was equipped with a medium-energy general purpose collimator (MEGP). The energy windows (EW) of ^177^Lu photopeaks were set at 113 keV ± 7.5% and 208.4 keV ± 7.5%. Images were acquired in step and shoot mode, with 32 × 2 views at 30 s per view. SPECT projections were reconstructed using an iterative algorithm with compensations for attenuation from CT images, scatter and full collimator-detector response in the Siemens E-Soft workstation (Syngo, MI Application version 32B, Siemens Medical Solution, Germany) with Flash 3D iterative algorithm (10 iterations; 8 subsets; Gaussian filter with 4.8 mm cut-off). As regards the scatter correction, the TEW (Triple Energy Window) correction was employed for the lower photopeak. The lower scatter window was set in the range from 87.58 to 104.53 keV (using a default window weight of 0.50), while the upper scatter window from 121.47 to 130.51 keV (using a default window weight of 0.94). With respect to the higher energy photopeak, the DEW (Double Energy Window) correction was employed and the lower scatter window ranged from 171.60 to 192.40 keV (using a default window weight of 0.75).

The FWHM of the system was measured by Grassi et al. [25] and the result was 10.4 ± 0.7 mm.

The imaging protocol consisted of four sequential SPECT/CT scans of the abdomen typically at 1, 24, 44 and 72 h p.i. (post injection). If necessary, also the thorax area at 1, 24 and 72 h p.i. was scanned.

A total of 141 lesions in the abdomen and a total of 13 lesions in the thorax were analysed.

### Dosimetry workflow

At the first cycle, a complete dosimetric evaluation of the selected tumours was performed based on SPECT/CT acquisitions. The SPECT/CT system was previously calibrated using a cylindrical Jasczcak phantom (Data Spectrum Corporation, USA) filled with a homogenous ^177^Lu radioactive solution. A calibration factor (CF = 36.5 cps/MBq) was determined by the ratio between the known activity and the measured total counts, following the procedure described by Grassi et al. [26]. A series of sequential multiple acquisitions of the phantom was performed. The standard uncertainty from repeating activity measurements was taken.

Subsequent to each SPECT acquisition, a CT image was acquired for attenuation correction. For radiation protection of patients, low resolution CT scans were acquired (90 mAs at the first scan and 30 mAs at the following acquisitions) and no contrast medium was used. As a consequence, most of the lesions were not visible on the CT image. For that reason, all tumours were manually segmented on the SPECT image. Contouring was performed in the Velocity Workstation (Varian Medical System, USA) using a variable threshold (as a percentage of the maximum value) defined by a nuclear medicine physician. To minimize misregistration errors, contours were outlined on the fused SPECT/CT image acquired at 24 h p.i., transferred automatically to all co-registered images and manually translated by the user, in case of need, to adapt them to the lesion site.

Activities were corrected for partial volume effects using recovery coefficients (RCs) previously determined based on phantoms with spherical inserts [27]. RCs as a function of insert volume were fitted with the following exponential curve:

1 $$ RC\left(\upsilon \right)=\alpha \cdot \exp \left(-\beta \cdot \upsilon \right)+\gamma $$

where *α*, *β* and *γ* are the fitting parameters and *v* (volume) is the independent variable.

Two different exponential curves were used to fit the time-activity points:

2 $$ {f}_1(t)={A}_0\cdot \exp \left(-{\lambda}_1\cdot t\right) $$

3 $$ {f}_2(t)={A}_0\cdot \mathit{\exp}\left(-{\lambda}_1\cdot t\right)\left[1-\mathit{\exp}\left(-{\lambda}_2\cdot t\right)\right] $$

where *A*_0_, *λ*_1_ and *λ*_2_ are the fitting parameters and *t* (time) is the independent variable. Equation 2 was used in case of monotonically decreasing data points or if only 3 time-points were available. Otherwise, Eq. 3 was used.

Time-integrated activities (TIAs) were calculated by solving the integral of the exponential functions, based on the fitting parameters.

**Fig. 1:**
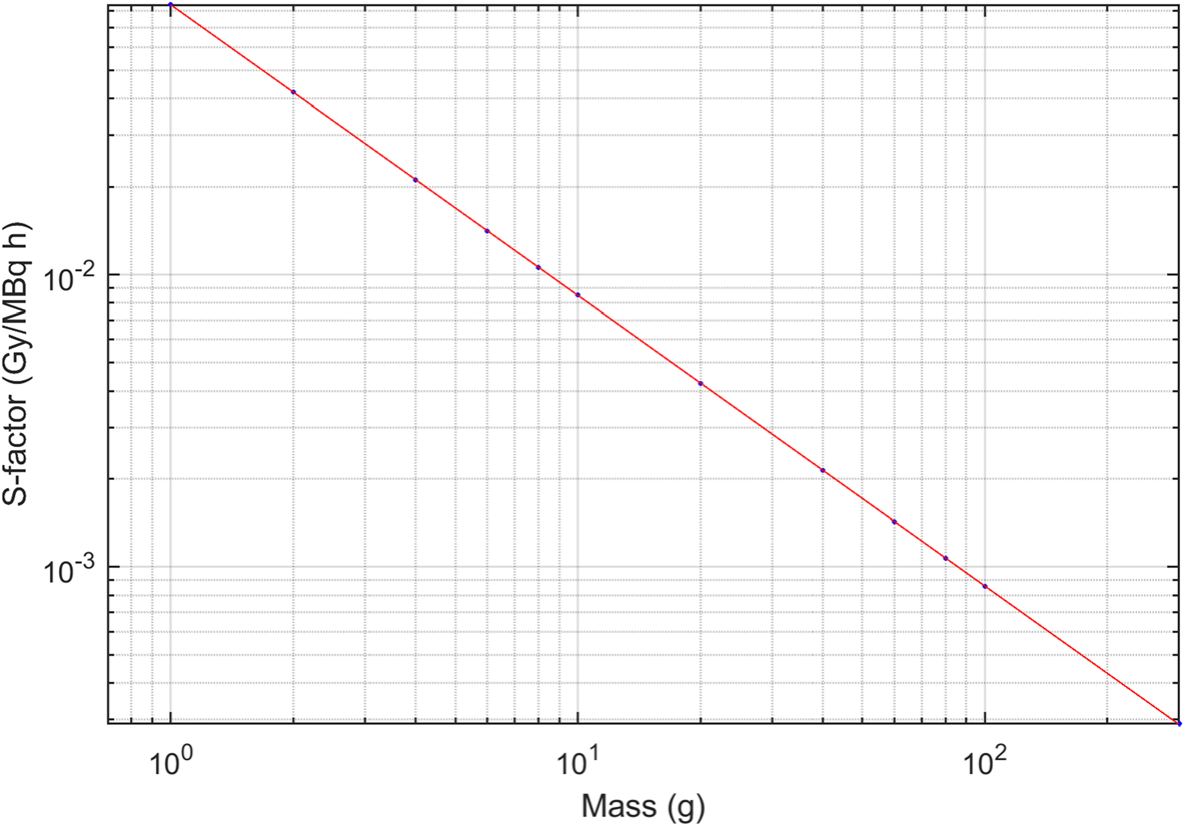
S-factors against mass for unit density spheres

Tumour absorbed doses were calculated using the OLINDA1.1 sphere model. S-factors derived from OLINDA1.1 were fitted against mass using a power function, as shown in Fig. 1. In this study, the absorbed dose at the first therapy cycle and the relative uncertainty was calculated.

### Uncertainty analysis

In this section, it is briefly described how uncertainty associated with each parameter within the dosimetry workflow was calculated. Please refer to the EANM published guidelines [24] for more details.

- Volume uncertainty *u*(*v*) was calculated using the analytical expression:

4 $$ {\left[\frac{u\left(\upsilon \right)}{\upsilon}\right]}^2={\left[3\frac{u(d)}{d}\right]}^2 $$

where *d* is the equivalent diameter of the outlined lesion, with uncertainty:

5 $$ {u}^2(d)=\frac{a^2}{6}+\frac{{\left(\mathrm{FWHM}\right)}^2}{4\ln 2} $$

where *a* is the voxel size and FWHM is the resolution of the imaging system.

- Referring to Eq. 1 and assuming the vector ***b*** = (*α*, *β*, *γ*, *v*)^*T*^, the squared standard uncertainty associated with RC was calculated as:

6 $$ {u}^2\left(\mathrm{RC}\right)={\mathbf{g}}_{\boldsymbol{b}}^T{\boldsymbol{V}}_{\boldsymbol{b}}{\mathbf{g}}_{\boldsymbol{b}} $$

where ***g***_***b***_ is the vector containing the partial derivatives of the first order of RC with respect to ***b*** and ***V***_***b***_ is the covariance matrix extended by one element, namely the partial derivative of the first order of *RC*(*v*) with respect to *v*.

- Uncertainty associated with the number of counts (C) within the VOI was calculated by assuming a Gaussian profile of the counts with standard deviation *σ* and by propagating the volume, CF and RC uncertainties:

7 $$ u(C)=\frac{C}{2\cdot RC}\cdot \frac{u\left(\upsilon \right)}{\upsilon}\left[\operatorname{erf}\left(\frac{2r}{\sigma \sqrt{2}}\right)-\frac{2\sigma }{r\sqrt{2\pi }}\left(1-{e}^{-\frac{2{r}^2}{\sigma^2}}\right)\right] $$

where *r* is the equivalent radius, erf is the error function and $\sigma =\frac{\mathrm{FWHM}}{2\sqrt{2\mathit{\ln}}2}$.

- Calibration factor uncertainty was determined by applying the LPU of measured activity (nominal accuracy of dose calibrator) and counts (standard deviation from multiple measurements).
- Uncertainties associated with the measured activities were determined by error propagation of CF, RC and C (uncertainty of administered activity was assumed to be negligible).
- Uncertainty associated with the fitting parameters (Eqs. 2 and 3) were assumed to be the main source of uncertainty of the time-activity curve fitting.
- Uncertainty associated with the TIA (denoted as $\overset{\sim }{A}$) included both the uncertainty of the fitting parameters and the uncertainty of the activities:

8 $$ {u}^2\left(\tilde{A}\right)={\mathbf{g}}_{\boldsymbol{b}}^T{\boldsymbol{V}}_{\boldsymbol{p}}{\mathbf{g}}_{\boldsymbol{p}}+{\left[\frac{u(A)}{A}\tilde{A}\right]}^2 $$

where ***g***_***p***_ is the gradient matrix of $$ \overset{\sim }{A} $$ with respect to the vector ***p*** containing the fitting parameters, ***V***_***p***_ is the covariance matrix and $$ \raisebox{1ex}{$u(A)$}\!\left/ \!\raisebox{-1ex}{$A$}\right. $$ is the fraction standard uncertainty associated with the measured activities.

- Uncertainty associated with S-factors, *u*(*S*), was derived by the propagation of the volume error (errors associated with the S-factors against volume fitting parameters were assumed to be negligible).
- Absorbed dose uncertainty was determined by applying the LPU:

9 $$ {\left[\frac{u(AD)}{AD}\right]}^2={\left[\frac{u\left(\tilde{A}\right)}{\tilde{A}}\right]}^2+{\left[\frac{u(S)}{S}\right]}^2+2\frac{u\left(\tilde{A},S\right)}{\tilde{A}\cdot S} $$

where $u\left(\overset{\sim }{A},S\right)$ is the covariance between $\overset{\sim }{A}$and *S*.

In addition to the parameters along the dosimetry workflow, the absorbed dose rate (AD rate) was calculated as the product between activity and S-factor. Absorbed dose rate uncertainty was determined using the following formula:

10 $$ {\left[\frac{u\left(\mathrm{AD}\;\mathrm{rate}\right)}{\mathrm{AD}\;\mathrm{rate}}\right]}^2={\left[\frac{u(A)}{A}\right]}^2+{\left[\frac{u(S)}{S}\right]}^2+2\frac{u\left(A,S\right)}{A\cdot S} $$

where u(A, S) is the covariance between *A* and *S*.

### Data analysis and statistics

All absorbed dose calculations and statistical analyses were performed in MATLAB R2019a (The MathWorks Inc., USA). A MATLAB script was developed and used to automatically calculate the uncertainty associated with each parameter within the absorbed dose calculation chain.

Box-plots were used to visualize the distribution of standard uncertainty of each variable included in this analysis. Association between each variable and the absorbed dose uncertainties were qualitatively assessed graphically.

As discussed in the EANM guidance, uncertainty in the absorbed dose is expected to largely depend on the precision with which the lesion volume can be estimated. An absorbed dose uncertainty (AD uncertainty) curve against lesion volume (v) was determined by the least squared fitting. A Power function of Eq. 6 was used to fit the empirical data points:

11 $$ \mathrm{AD}\;\mathrm{uncertainty}\kern0.5em \left(\upsilon \right)=A\cdot {\upsilon}^B $$

where A and B are the fitting parameters and *v* is the independent variable.

Absorbed dose rate uncertainty against tumour volume was also plotted on a graph in order to assess the relationship between these quantities.

In this study, we further evaluated the expected improvement in absorbed dose uncertainty attainable with potential improvement in the accuracy of the volume estimation. This was achieved by repeating all uncertainty calculations assuming a range of system spatial resolutions, such as those typical of ^68^Ga PET/CT and CT imaging.

## Results

Forty-nine patients (22 males, 27 females, median age 62 years, range 36–79 years) were treated with PRRT. Among the 154 lesions analysed, 100 were situated within the liver (64.9%), 8 in the pancreas (5.2%), 5 in the lung (3.2%), 18 were bone lesions (11.7%), 18 were lymph nodes (11.7%) and 5 were in other locations (3.2%).

The median value of the contoured volumes on SPECT images was 6.9 mL and the interquartile range was 4.7–17.2 mL.

**Fig. 2:**
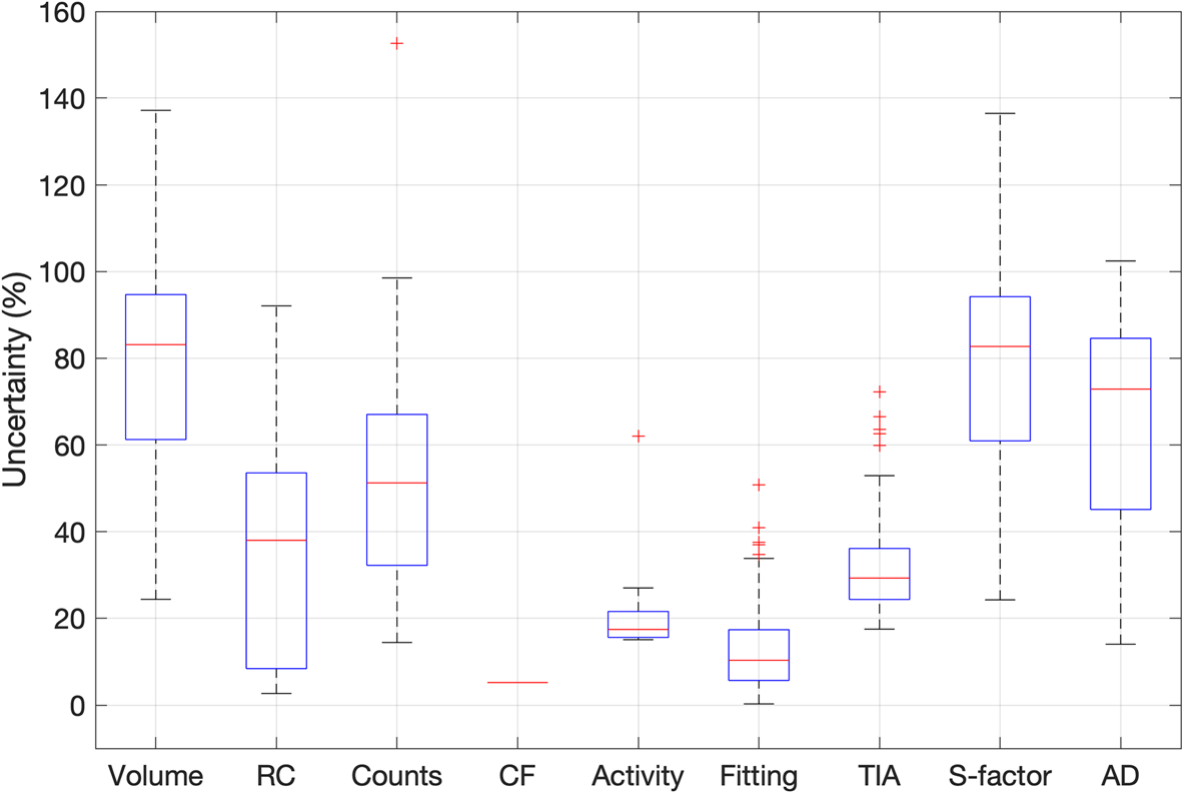
Distribution of uncertainty (%) for each step of the absorbed dose calculation schema

**Fig. 3:**
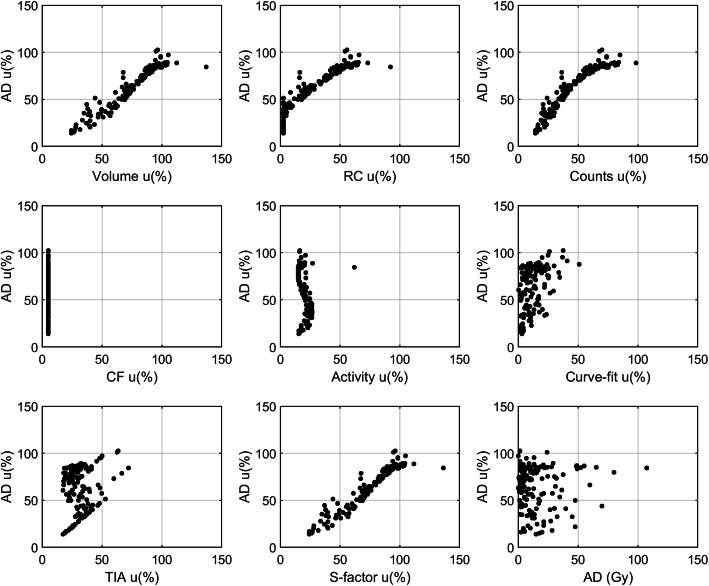
Relationship between absorbed dose uncertainty (*y*-axis) and volume, RC, counts, CF, activity, curve fitting parameters, time-integrated activity and S-factors uncertainties (*x*-axis). The graph at the bottom right shows the absorbed dose (Gy) against the absorbed dose uncertainty

The average uncertainty in absorbed dose was relatively high with a mean of 65% and a median value of 73%. A wide range of uncertainty values was observed (14–102%). Figure 2 shows the distribution of the relative uncertainty for each parameter calculated along the dosimetry chain. The highest relative uncertainties were found to be associated with volume and S-factor. It is worth noting that S-factor uncertainty is strictly dependent on the volume uncertainty, which can be considered as a primary source of error. The absorbed dose uncertainty was plotted against the uncertainty associated with each of the parameters along with the dosimetry workflows, as shown in Fig. 3. Different patterns were obtained for each variable, demonstrating the complex relationship that each quantity has on the estimate of absorbed dose. A clear relationship was observed between absorbed dose uncertainty and volume uncertainty, as evidence that the major factors affecting the accuracy of absorbed dose calculation originate from the volume delineation.

**Fig. 4:**
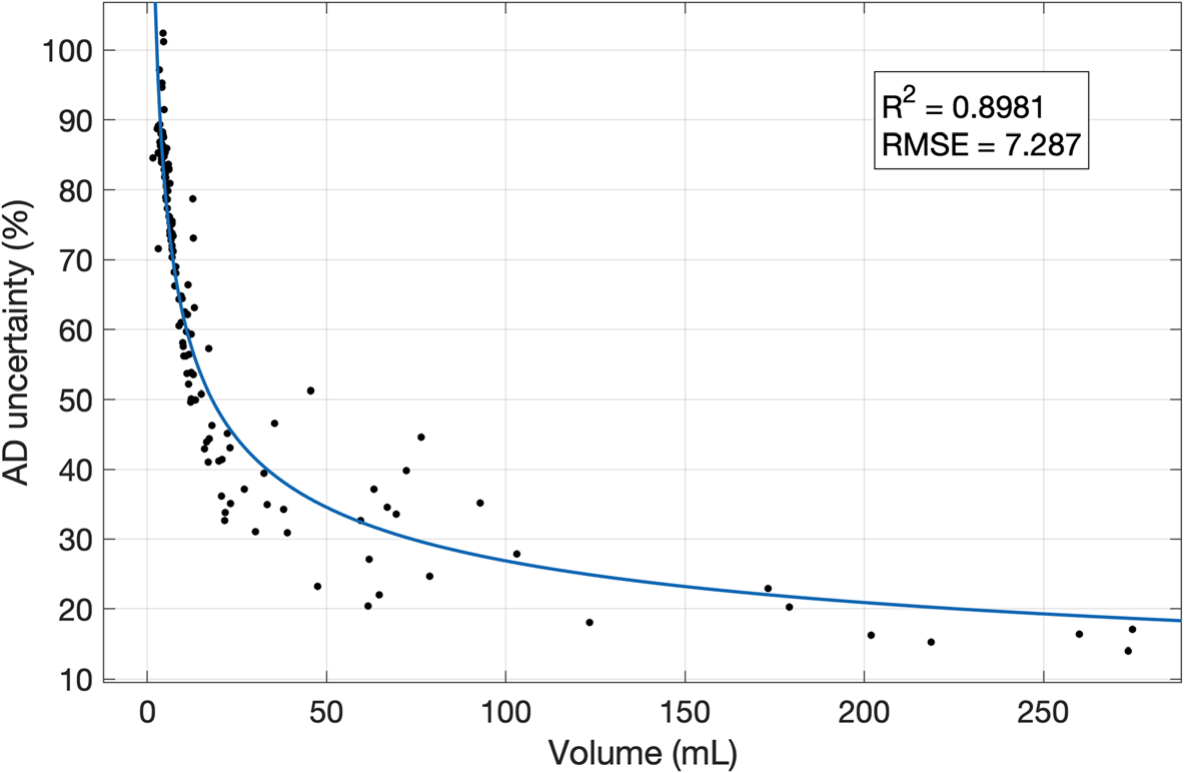
Absorbed dose uncertainty (%) against volume (mL). Points were fitted with a power function. R2 and RMSE are reported in the graph

The absorbed dose uncertainties against volume were fitted using the power function of Eq. 11. The fit coefficients are shown in Table 1, while the fit curve is shown in Fig. 4.

**Table 1:** Power curve best-fit parameters of absorbed dose uncertainty against volume

	Value	Confidence interval (95%)
A	142.9	(135.9, 149.8)
B	− 0.36	(− 0.39, − 0.34)

**Fig. 5:**
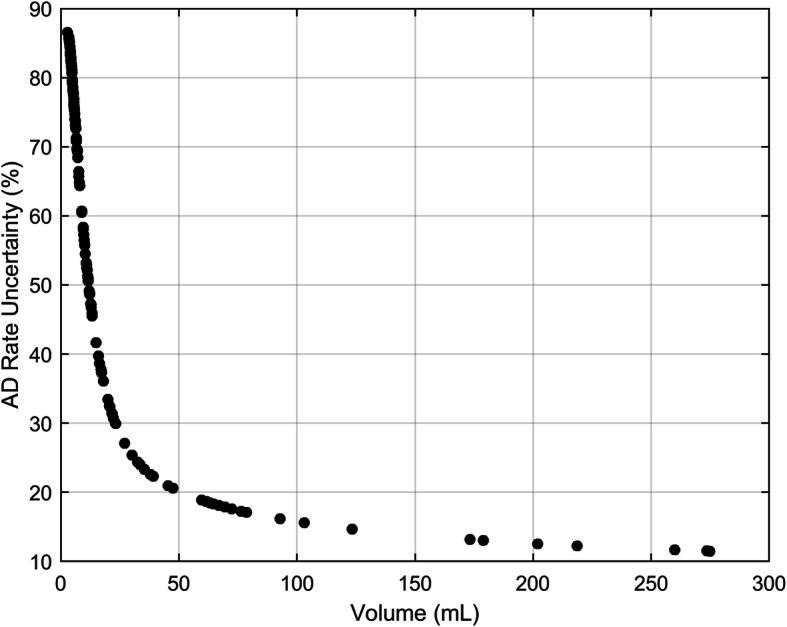
Absorbed dose rate uncertainty (%) against volume (mL)

The average absorbed dose rate relative uncertainty was 58%, with a median value of 69% and values ranging between 11% and 87%. Absorbed dose rate uncertainty against volume is shown in Fig. 5.

In order to assess if the number of data-points affects the accuracy of the time-activity curve (TAC) fitting, patients with four time-points and patients with three time-points were separately evaluated. A total of 141 four-point datasets and 13 three-points datasets were analysed, with an average of 12% relative uncertainty of TAC fitting on the former and an average of 16% on the latter.

**Fig. 6:**
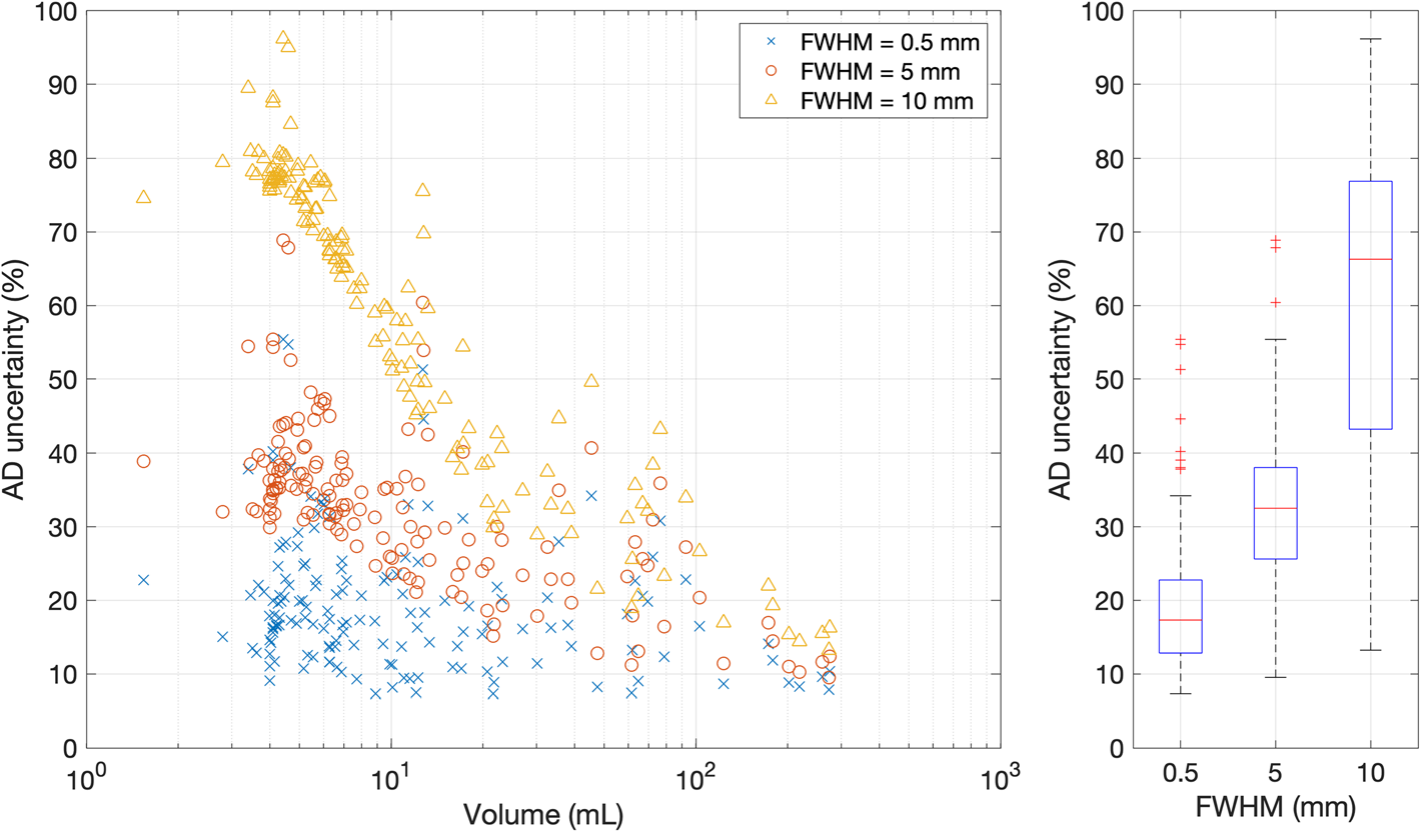
On the left, absorbed dose uncertainty (%) against volume (mL) calculated for all the lesions, postulating imaging systems with FWHM equal to 0.5, 5 and 10 mm (representative of CT, PET and SPECT systems, respectively). On the right, distributions of absorbed dose uncertainty (%) for each value of the FWHM

**Fig. 7:**
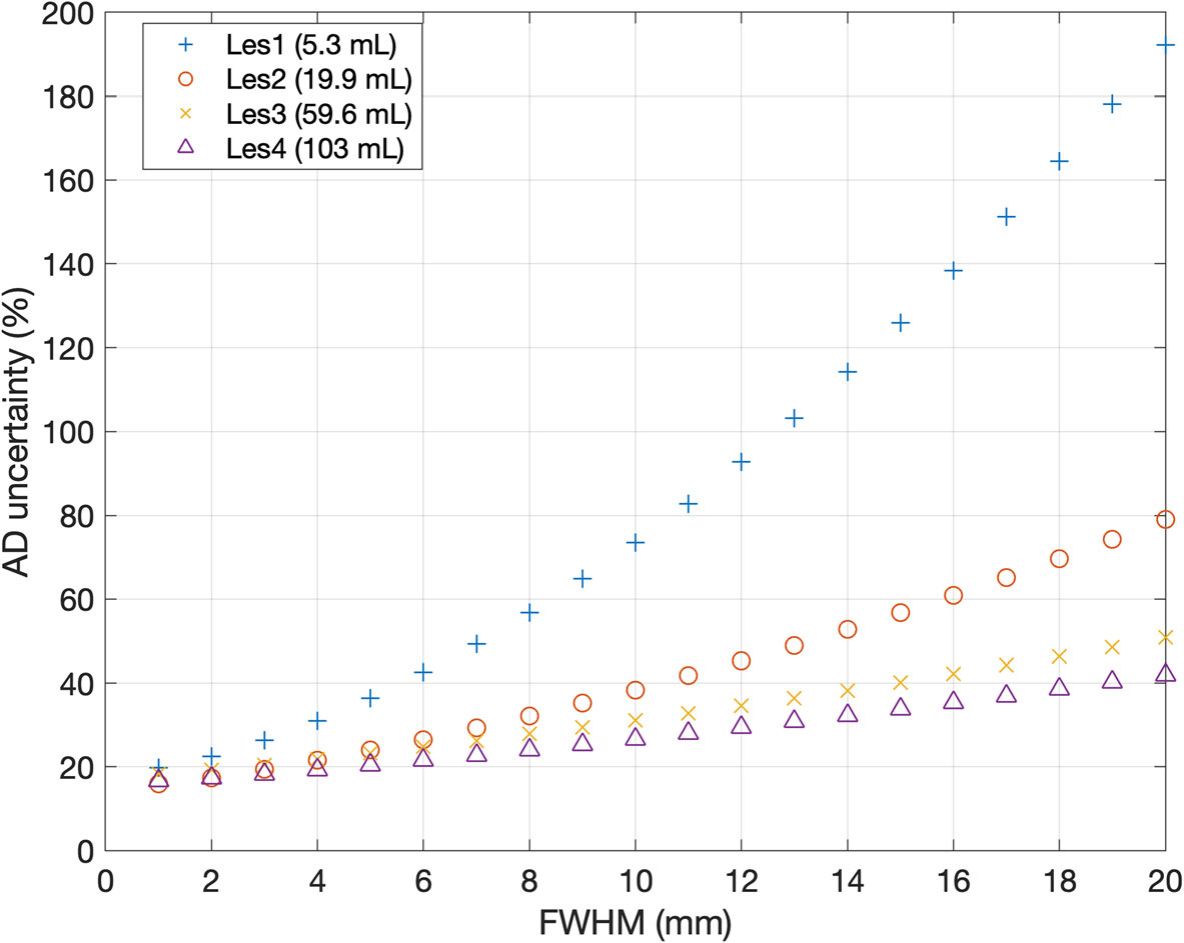
Absorbed dose uncertainty (%) as a function of the imaging system spatial resolution (FWHM in mm) in four lesions. Lesions were chosen to fill a range of different values of volume

**Fig. 8:**
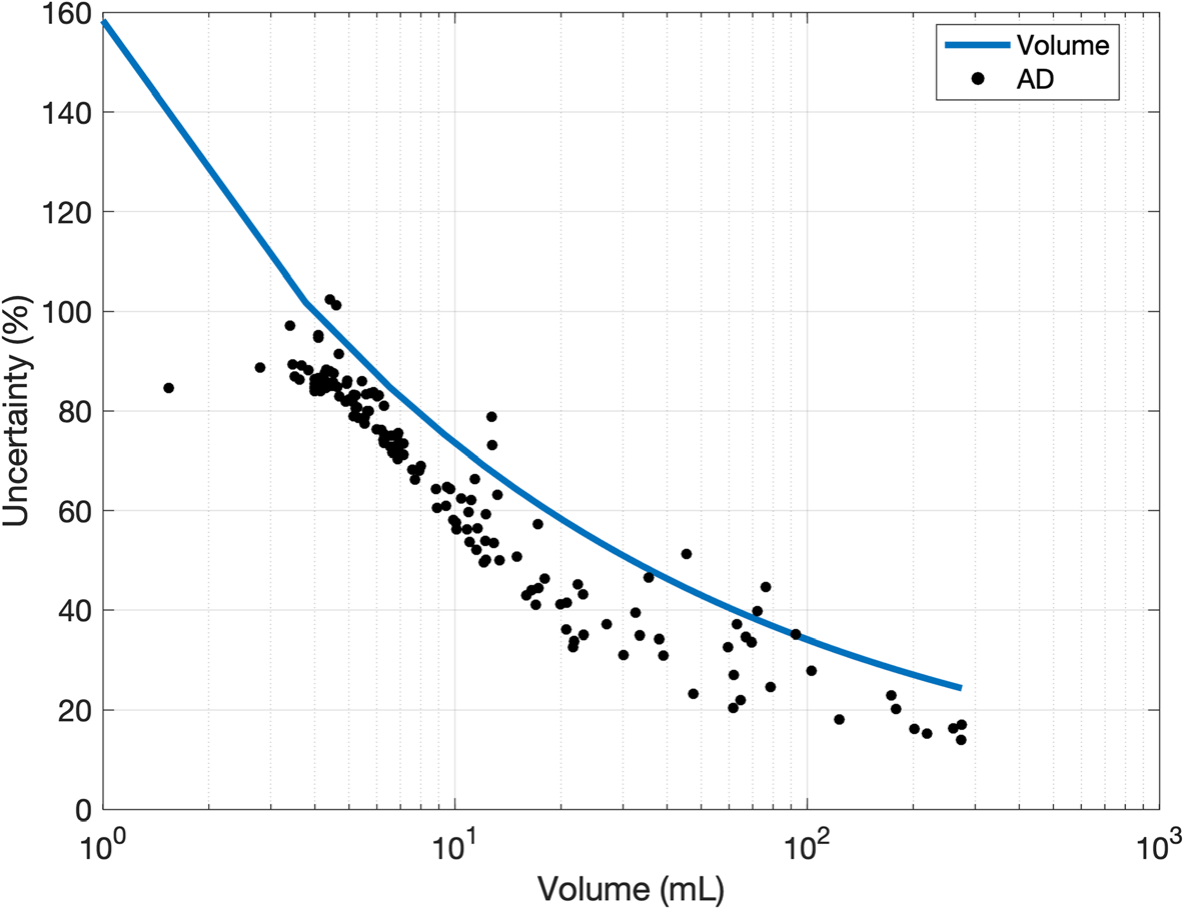
Absorbed dose uncertainty (black points) and the volume uncertainty (blue line) as a function of the delineated VOI volume

The effect of different values of spatial resolution was investigated by hypothetically changing the value of FWHM given in Eq. 5. Figure 6 shows the relative absorbed dose uncertainty re-calculated for all the lesions, assuming three different values (0.5, 5 and 10 mm) of FWHM (note: the actual FWHM of the acquisition system was 10.4 mm). These values were chosen to represent the typical spatial resolution of CT, PET and SPECT acquisition systems, respectively. In Fig. 7, four lesions with very different volumes were considered and the absorbed dose uncertainty was estimated for a range of values of the system spatial resolution. Absorbed dose uncertainty and volume uncertainty were plotted on the same axis against volume in Fig. 8 to further assess the relationship between these variables.

## Discussion

Lack of knowledge of absorbed dose calculation uncertainties has been a factor that has impeded widespread uptake of dosimetry in MRT.

The EANM guidelines provide a schema of uncertainty propagation to evaluate the standard uncertainty in absorbed dose to a target. This schema was based on the recommendations described within the GUM [28] and necessarily involves the formation of covariance matrices for several steps of the dosimetry process. In this work, we have applied the EANM guidelines to evaluate the uncertainty of tumour dosimetry calculations in PRRT. This study carried out, for the first time, the uncertainty analysis of the entire process of dosimetry calculation on a large sample of clinical cases compared to the existing scientific context. A total of 154 lesions were analyzed.

As shown in Fig. 2, the fractional uncertainty associated with the considered quantities (volume, CF, S-factor, etc.) was widespread around the median value, incurring a high inter-lesion variability. Volume and S-factor are the quantities with the highest values of uncertainty associated. It is worth noting that S-factor uncertainty mainly originates by the volume uncertainty and, in that sense, it is not a primary source of uncertainty. These results confirmed that the accuracy in absorbed dose and absorbed dose rate is dominated by the accuracy in the delineation of the VOI. For example, when contouring a volume, the uncertainty in edge definition due to the limited spatial resolution, together with the voxel width, involves errors in the assessment of the volume.

The uncertainty associated with the volume is then propagated to many of the other parameters (RC, counts, activity, fitting, TIA, S-factor and absorbed dose). The relationship between fractional absorbed dose uncertainty and tumour volume is evident in Fig. 4. The analytical power model for this relationship fitted the empirical data points well, and this could be useful, in clinical practice, for a quick estimate of uncertainty without implementing the entire error propagation schema, which could be useful to select the lesions to be monitored for patient outcome assessment.

Figure 8 shows the uncertainty in absorbed dose (black points) and the uncertainty in volume (blue line) on the same axis. This graph shows the “weight” of the uncertainty associated with the volume segmentation and other parameters on the accuracy of the dosimetric calculation. From a practical point of view, from Fig. 8, it is possible to deduce that uncertainty pertaining to a smaller lesion is mainly due to the volume delineation. For larger lesions, volume contouring impact is less significant and other parameters, such as random effects affecting the confidence of the fit parameters for the TAC, begin to dominate. As a result, data-points are increasingly distributed beyond the empirical function as the volume increases. It is also interesting to note that the fractional uncertainty in absorbed dose is lower than that of the volume uncertainty as covariance effects within the dosimetry chain reduce the overall uncertainty in absorbed dose. The random component of the fitting parameters does not contribute to the absorbed dose rate uncertainty. This results in smaller fluctuations of data-points around the average value, as evident by comparing Fig. 5 with Fig. 4. The accuracy of time-activity curve fitting depends on the number of data-points, the scan times and the theoretical model function employed. The optimal scan times to perform dosimetry in PRRT are yet to be determined. Sandstrom et al. [29] proposed to use a late time-point at 7 days, the EANM dosimetry committee [30] suggested to use at least three time-points, while Del Prete et al. [31] and Hänscheid et al. [32] proposed simplified dosimetry protocols based on two time-points and one time-point, respectively. It is evident that the greater the number of time-points, the more uncertainty will be reduced. However, an evaluation in terms of cost/benefits should be performed to determine the optimal solution. The average ^177^Lu-DOTATATE tumour effective half-life was found to be between 77 h and 110 h [31–33]. As a consequence, tumour uptake at 70 h p.i. (last acquisition time-point based on our clinical protocol) is about 60% of the maximum value. Extrapolation of the simulated curve beyond the last acquisition point inherently leads to errors on the calculation of absorbed dose, since the lesion-specific effective half-life may be misestimated. Half-life, in fact, depends on the tumour biology through the biological half-life. Biology influences and may cause the differentiation of the retention in each lesion, even in the same patient. However, restrictions on the timing and number of samples are necessary for patient benefit and extrapolation of the simulated curve to time intervals for which no data exist needs to be performed. Since no specific information about the lesion biological retention is available in practice, uncertainty of the time-integrated activity is estimated based on the goodness of the analytical function to fit the data. In this study, 141 tumours with four time-points (1, 24, 40 and 70 h p.i.) and 13 tumours with three time-points (1, 24, 70 h p.i.) were analyzed. Our data suggest that the use of four time-points reduces the uncertainty of the TAC fitting by 4% compared to using three time-points (12% to 16%). Moreover, it should be noted that it is undesirable to fit three data-points with a three parameters bi-exponential curve like the one in Eq. 3. From a pure mathematical point of view, this will result in an unreliable model with no test of goodness of fit and so with no possibility of checking the parameters. However, with early time-points acquisition (1 h p.i.), we often saw evidence of the initial uptake phase before the time-activity curve started to decrease in mono-exponential washout. Hence, using a mono-exponential curve to model the time-activity decay may not be the optimal choice. The potential impact on the accuracy of absorbed dose needs to be investigated; however, it goes beyond the aim of this study. In this work, time-activity points were fitted by using either mono- or bi-exponential curves. The optimal fit function should be chosen for each case based on the number and the distribution of available data-points, possibly by using model selection criteria as discussed by Kletting et al. [34].

These results may be useful to provide the user with an indication about the typical expected uncertainty while performing dosimetry. Assuming an acceptable absorbed dose uncertainty of 40% as a reference, which is the typical absorbed dose uncertainty on clinical cases as reported by Grassi et al., the correspondent cut-off tumour volume is around 33 mL. Consequently, it can be concluded that absorbed doses to lesions with volumes smaller than 33 mL cannot be determined to a sufficient level of confidence to make the result meaningful. However, it should be noted that these values depend on the spatial resolution of the imaging system and on the method used to contour the VOI. In this study, the VOIs were manually contoured on the SPECT images.

Figures 6 and 7 show the impact of spatial resolution on final uncertainty evaluated by postulating a different FWHM for each of the imaging systems. These results have demonstrated that uncertainty would be significantly reduced by increasing the spatial resolution. This effect would be particularly significant in the case of small volumes. Hence, a minimal acceptable volume cut-off should be set, depending on the spatial resolution of the system available in the site. A standard gamma camera, combined with an iterative reconstruction algorithm that includes attenuation, scatter and collimator-detector response, provides images with a spatial resolution around 1 cm for ^177^Lu, as reported in [25].

In this study, 128 lesions (out of 154) had a volume smaller than 33 mL. All 154 lesions were considered of clinical importance in the trial and were used in the treatment planning. It should be noted that for this analysis, a tumour volume cut-off was not introduced (consequently also lesions with very small volumes were analysed) to provide worthy results in the whole clinical range of volumes.

Anyway exclusion of tumours below 33 mL is undesirable as they may be of clinical importance; rather, the improvement of spatial resolution and VOIs delineation is desirable. The accuracy of VOI delineation may be improved by using the appropriate acquisition/reconstruction protocol (accounting for acquisition statistics, matrix, collimator type, reconstruction settings) to obtain images with a spatial resolution as high as possible. Lesions may be delineated using contrast-enhanced CT or ^68^Ga-PET where feasible, which are characterized by a better spatial resolution than SPECT imaging. Contouring on images with a spatial resolution of 5 mm (typical of PET images or new generation SPECT systems) would provide a cut-off tumour volume of 4 mL (considering an absorbed dose uncertainty equal to 40%). Almost all the lesions provided absorbed dose uncertainty smaller than 40% if a spatial resolution equal to 0.5 mm (typical of CT images) was used. In that case, an absorbed dose uncertainty cut-off lower than 40% may be set in order to increase the significance of absorbed dose calculations. For example, a cut-off volume of 4 mL would provide a confidence level of absorbed dose calculation around 20%. However, the possibility of using CT in place of SPECT or PET is to be evaluated, maybe combining both the morphological and functional information. It is worth to be noted performances of imaging systems are rapidly improving and new generation cameras provide images with better spatial resolution. Images with 5 mm of spatial resolution are in the present day within the reach of the most advanced SPECT/CT systems and even better resolutions may be reached with PET/CT systems. Uncertainty of volume evaluation might be further reduced by averaging VOIs delineated by different operators. However, this approach may be difficult to be applied in clinics.

This study had some limitations because some sources of uncertainty were not included in this analysis. VOIs were outlined using a standard threshold when possible; however, in some cases, the threshold was adapted by the physicians in order to adequately contour the tumour volume in relation to the tumour uptake and the activity of the surrounding tissues. For that reason, the uncertainty of volume determination is operator-dependent, but in this study, that component was not taken into account. Errors due to image misregistration were not included in this analysis. Misalignment of VOIs with the tumours was assumed to be negligible as each VOI was visually checked and manually adjusted in case of need. Activities were corrected for partial volume effect using pre-calculated RCs based on phantom measurements. This method makes some approximations: it is assumed lesions to have a spherical shape and counts do not spill-in from surrounding tissues. These approximations affect the accuracy of partial volume effect correction; however, they were not considered in this study. Following the MIRD schema, it was assumed that the tumour tissue was homogeneous, the tumours had spherical shapes and the target volumes were the sources activity volumes (i.e. the contribution of absorbed dose from the surrounding organs was not considered). There are uncertainties associated with deviations between these assumptions and reality, but they are outside the scope of this framework.

## Conclusion

In conclusion, this study provided the first analysis of uncertainties of tumour absorbed dose calculations on a sample of clinical cases treated with PRRT. Assessment of uncertainties provides the degree of consistency of the data and allows to adequately weigh results in treatment planning. For that reason, it is firmly recommended to include the analysis of uncertainty for any measured or calculated parameters in clinical routine. However, such analyses in MRT are rarely performed. The application of uncertainty analysis in clinical practice may help clinicians to select tumours for treatment response evaluation and may help to identify parameters that more affect the accuracy of calculation. Such analysis may increase the validity of dosimetry, and in turn, it would encourage physicians to use dosimetry in treatment planning. In the research field, it may facilitate the determination of the dose-response relationship and it may allow to compare results among different clinical sites. This study showed volume delineation to be one of the parameters which more affect the accuracy of absorbed dose calculations and it most likely is the easiest side to ameliorate in the clinical practice. Based on these results, using PET or CT imaging or new generation SPECT systems would reduce the amount of uncertainty by a factor between 50% and 70% in comparison to using SPECT images acquired with less recent scanners. The ability to improve the accuracy of absorbed dose calculations might be crucial to optimize treatment efficacy in internal radionuclide therapy.

## Data Availability

The datasets used and/or analysed during the current study are available from the corresponding author on reasonable request.

## Abbreviations

AD: Absorbed dose
CF: Calibration factor
CT: Computed tomography
EANM: European Association of Nuclear Medicine
EW: Energy window
FWHM: Full width at half maximum
LPU: Law of propagation of uncertainty
MRT: Molecular radiotherapy
NET: Neuroendocrine tumours
PET: Positron emission tomography
PRRT: Peptide receptor radionuclide therapy
RC: Recovery coefficient
SPECT: Single-photon emission computed tomography
TAC: Time-activity curve
TIA: Time-integrated activity
VOI: Volume-of-interest
